# Reverse PCA, a Systematic Approach for Identifying Genes Important for the Physical Interaction between Protein Pairs

**DOI:** 10.1371/journal.pgen.1003838

**Published:** 2013-10-10

**Authors:** Ifat Lev, Marina Volpe, Liron Goor, Nelly Levinton, Liach Emuna, Shay Ben-Aroya

**Affiliations:** Faculty of Life Sciences Bar-Ilan University, Ramat-Gan, Israel; University of Ottawa, Canada

## Abstract

Protein-protein interactions (PPIs) are of central importance for many areas of biological research. Several complementary high-throughput technologies have been developed to study PPIs. The wealth of information that emerged from these technologies led to the first maps of the protein interactomes of several model organisms. Many changes can occur in protein complexes as a result of genetic and biochemical perturbations. In the absence of a suitable assay, such changes are difficult to identify, and thus have been poorly characterized. In this study, we present a novel genetic approach (termed “reverse PCA”) that allows the identification of genes whose products are required for the physical interaction between two given proteins. Our assay starts with a yeast strain in which the interaction between two proteins of interest can be detected by resistance to the drug, methotrexate, in the context of the protein-fragment complementation assay (PCA). Using synthetic genetic array (SGA) technology, we can systematically screen mutant libraries of the yeast *Saccharomyces cerevisiae* to identify those mutations that disrupt the physical interaction of interest. We were able to successfully validate this novel approach by identifying mutants that dissociate the conserved interaction between Cia2 and Mms19, two proteins involved in Iron-Sulfur protein biogenesis and genome stability. This method will facilitate the study of protein structure-function relationships, and may help in elucidating the mechanisms that regulate PPIs.

## Author Summary

Protein–protein interactions (PPI) occur when two or more proteins bind together to form large molecular machines. The importance of PPIs led to the development of multitude technologies to detect them, and to the first maps of the protein interactomes. One important challenge in biology is to understand how protein complexes respond to genetic perturbations; however, in the absence of a suitable assay, such changes have been poorly characterized. Here, we present a novel systematic genetic approach (termed “reverse PCA”), that demonstrates how the yeast protein complementation assay (PCA), coupled with the synthetic genetic array (SGA) technology may be used to study the modulation of protein–protein interactions *in-vivo* in response to genetic perturbations. Our assay starts with a yeast strain in which the interaction between given proteins can be detected by resistance to the drug, methotrexate. Using the SGA technology, we can systematically identify yeast mutants that reverse this interaction. We were able to successfully validate this approach by identifying mutants that dissociate the conserved interaction between Cia2 and Mms19, two proteins involved in Iron-Sulfur protein biogenesis and genome stability. This method will facilitate the study of protein structure-function relationships, and elucidate the mechanisms that regulate PPIs.

## Introduction

Protein-protein interactions (PPIs) are critical to virtually all biological processes, from the formation of cellular macromolecular structures and enzymatic complexes to the regulation of signal transduction pathways. Hence, detailed analysis of these interactions is essential for understanding biological phenomena. Many experimental methods have been developed in the past decade for mapping PPI networks. For eukaryotes, the most popular experimental platform for large-scale analysis of PPIs is the yeast, *Saccharomyces cerevisiae*. Protein complexes have been characterized in yeast using affinity purification followed by mass spectrometry (AP/MS) [1]. Other approaches such as high-throughput yeast two-hybrid (Y2H) analyses [2], fluorescence resonance energy transfer (FRET) [3], and protein-fragment complementation assay (PCA) [4] have been used to identify binary interactions. The systematic unbiased utilization of these methods led to various maps of the protein interactome of several model organisms [2], [5], [6].

There are many changes in protein complexes that occur as a result of genetic and biochemical perturbations. These include changes in protein levels and localization, and posttranslational modifications that may alter the bond between interacting partners (for more details see Figure S1). One drawback of the above-mentioned studies is that they were performed on a single genetic background, and thus, potential genetic modifications that can dissociate the interaction remain unidentified. The identification of trans-acting mutants that dissociate a particular PPI is valuable for unraveling important regulatory mechanisms, and for defining the biological effect of a specific perturbation. Despite the great importance of such data, none of the available experimental systems allow the systematic detection of such dissociation events. Rather, they are limited to positive selection of protein-protein association events.

PCAs are a family of assays for identifying interactions between protein pairs [7]. In this strategy, PPIs are measured by fusing each of the proteins of interest to complementary N- or C-terminal peptides of a reporter protein. Upon interaction of the two fusion proteins, the reporter protein fragments are brought into proximity, thus reconstituting the activity of the reporter, such that it provides a detectable signal [8]. PCAs have been created using many different reporter proteins and thus enable different types of readouts. A PCA based on a mutated version of the murine dihydrofolate reductase enzyme (mDHFR) was adapted to the yeast *Saccharomyces cerevisiae* [5], [9]. In this case, mDHFR is split into two complementary fragments (F[1,2], and F[3]) and inserted at the C-termini of the two genes of interest. The functional copy of the mDHFR confers resistance to the DHFR inhibitor, methotrexate (MTX), which inhibits the native *S. cerevisiae* DHFR. Thus, the interaction between the two proteins of interest can be detected as cell growth on media in the presence of MTX. This approach has been recently used to systematically identify nearly all possible binary combinations of yeast proteins, and has led to the identification of 2770 interactions that represent an *in vivo* map of the yeast PPI network [5].

In this report, we describe the “reverse-PCA” (rPCA) system, which combines the PCA and the synthetic genetic array (SGA) methodologies [10]. In the SGA approach a *MAT*α query strain carrying any genetic element (or any number of genetic elements) marked by a selectable marker(s) can be crossed to an ordered array of mutants collection (*MAT* **a**). The resulting array of heterozygous diploids can then be sporulated, and a set of desired *MAT* **a** haploid meiotic progeny cells can be subsequently selected, exploiting a cleverly designed SGA haploid selection marker (HSM) [10].

The combination of these approaches allows us to systematically detect trans-acting proteins that, when mutated, dissociate specific PPIs in *S. cerevisiae*. We demonstrate the feasibility of this approach by identifying previously characterized and novel proteins that dissociate the conserved interaction between Cia2 and Mms19, two proteins that play a role in Iron-Sulfur cluster biogenesis, and in genome stability [11], [12]. Our results validate this method as an efficient and scalable approach that is expected to promote biological discovery.

## Results/Discussion

### The systematic rPCA - A method for detecting trans-acting mutations dissociating a specific PPI in *S. cerevisiae*

**Figure 1:**
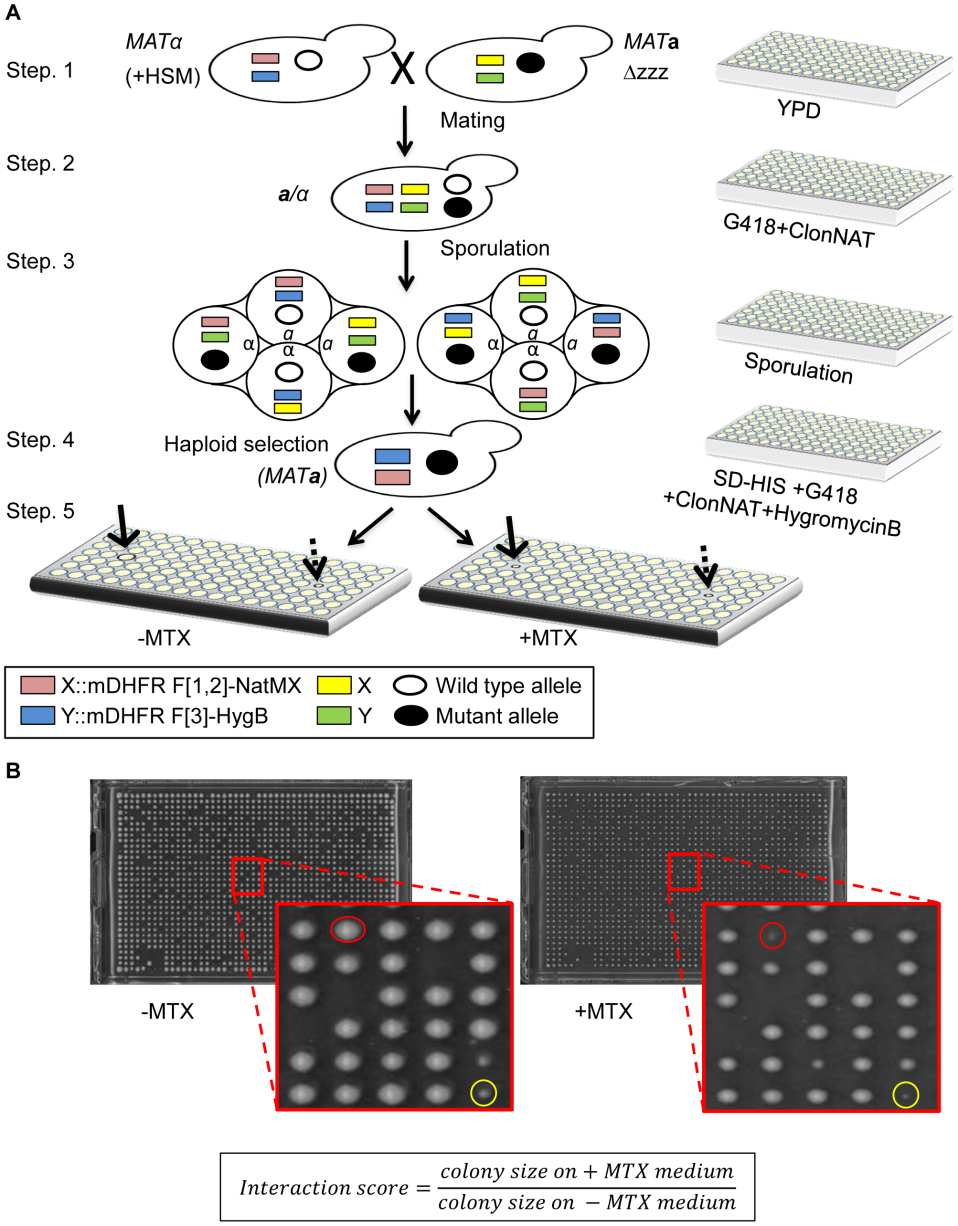
(A) Scheme of rPCA, a systematic method for detecting trans-acting mutations that dissociate a specific PPI in *S. cerevisiae*. *Step 1*. The *MAT*α query strain carries two complementary fragments of mDHFR (F[1,2], and F[3]), fused at the C-terminus of the proteins of interest (X and Y), and linked to dominant selectable markers, NatMX and HygB respectively (X::mDHFR F[1,2]-NatMX, and Y::mDHFR F[3]-HygB), which confer resistance to the antibiotics nourseothricin (ClonNAT), and HygromycinB (HygB). In addition, these strains also contain the MFA1pr-*HIS3*, *can1*Δ and *lyp1*Δ SGA haploid selection markers (HSM). The query strain is crossed to an ordered array of *MAT* **a** viable deletion mutants (*zzz*Δ), each carrying a gene deletion mutation linked to a *kanMX* marker, which confers resistance to the antibiotic Geneticin (G418) (illustrated here), or *MAT* **a** libraries of temperature sensitive alleles of most essential genes. *Step 2*. The growth of resultant zygotes is selected on medium containing ClonNAT and G418. *Step 3*. The heterozygous diploids are transferred to medium with reduced levels of carbon and nitrogen to induce sporulation and the formation of haploid meiotic spore progeny. *Step 4*. Spores are transferred to a haploid selection medium, i.e., synthetic medium lacking histidine, which allows for selective germination of *MAT* **a** meiotic progeny (since only these cells express the MFA1pr-*HIS3* reporter), and containing canavanine and thialysine, which allows for selective germination of meiotic progeny that carry the *can1*Δ and *lyp1*Δ HSMs. *Step 5*. The *MAT* **a** meiotic progeny are then transferred to haploid selection medium containing G418 (for growth of meiotic progeny that carry the gene deletion mutation), and HygB and clonNAT, which select for the fusion proteins. Medium lacking MTX (−MTX) (*left*), is used as a control, and provides an indication of mutants that affect growth rate *per-se* under normal conditions (indicated by a dashed black arrow). The haploids that were selected for further analysis showed impaired growth on the experimental MTX-containing medium (+MTX) (*right*), when compared to the control array (indicated by black arrows). **(B) Example of colonies that were selected for further analysis.** Representative images of colonies obtained 2 days after pinning of a single 1536-density array plate from step-5 (see Figure 1A). Example of a mutant that affects growth rate *per-se* under normal conditions (circled in yellow), and a mutant that specifically affects growth in the presence of MTX, and was selected for further analysis (circled in red). The formula shows the calculation that was used for the scoring of each colony.

In the yeast mDHFR PCA, the interaction between two proteins of interest (designated here as X and Y) allows yeast cells to grow in the presence of MTX. We designed a system to uncover mutants that impair the growth on MTX containing media. The query strain for the rPCA was chosen from a dataset of 2,770 interactions that were recently identified by mDHFR PCA [5]. In this strain, the two complementary fragments of mDHFR (F[1,2], and F[3]) are each fused to the C-terminal of X and Y (X-F[1,2], and Y-F[3]). Using SGA methodology [10], we crossed this strain to ordered arrays of three libraries which together encompass mutations in every yeast gene (Figure 1A, step-1). The first library was the yeast deletion library [13], which consists of ∼4500 strains, each harboring a deletion in a single non-essential gene. The second and third were two complementary libraries, which together consist of ∼1000 strains, each expressing a temperature sensitive (Ts) allele of an essential gene [14], [15]. The resulting array of heterozygous diploids was then induced to undergo meiosis (Figure 1A, step-2, 3), and the set of desired *MAT* **a** haploid meiotic progeny cells was subsequently selected, exploiting the SGA haploid selection marker (HSM) (Figure 1A, step-4). These steps allowed the recovery of a library of ∼6000 haploid meiotic progeny, each harboring both X-F[1,2], and Y-F[3] fusion proteins, on the background of a mutation in a single yeast gene. This array was used as a control, and provided samples of mutants that affect growth rate *per-se* under normal conditions (Figure 1A, step-5 (*left*), indicated by a dashed arrow). These haploids were also transferred to a second plate to select for MTX resistance. Colony growth was assessed using an automated computer-based scoring system. This system analyzes digital images of colonies to generate an estimate of the relative growth rate based on pixel density [16]. Impaired PPI was scored when the colony size on the MTX containing medium was significantly smaller than that on the control array (Figure 1A, step-5 (*right*), indicated by a black arrow, and Figure 1B).

**Figure 2:**
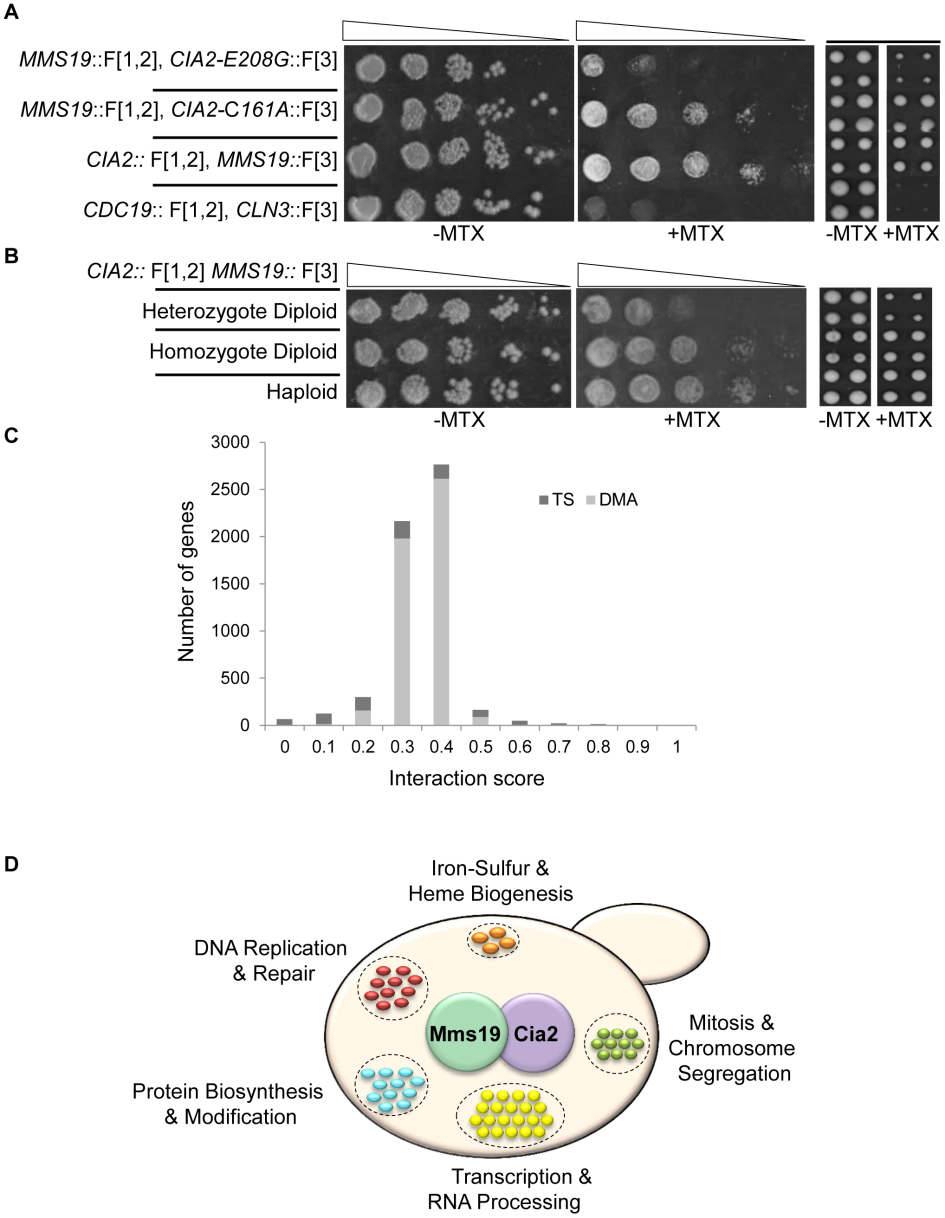
(A) PPI indicated by cell resistance to MTX can be reversed by *cis*-acting mutations. Ten-fold serial dilutions (*left*), or robotic pinning in quadruplicates of the corresponding strains (*right*), containing the interacting fusion proteins Cia2:: F[1,2]; Mms19:: F[3], on a rich medium lacking (control), or supplemented with 200 µg/ml MTX. *cia2-*E208G::F[3] represents a *cis*-acting point mutation in Cia2, that impairs the interaction with Mms19. The *cia2*-C161A::F[3] point mutation was used as a positive control. The pyruvate kinase *CDC19*::F[1,2], and the G1 cyclin *CLN3*::F[3] fusion proteins were used as negative controls. **(B) Growth on MTX is proportional to the level of complexes formed by two interacting proteins.** Similar to (A), the reduction in the relative abundance of reconstituted functional copy of mDHFR is indicated by using strains hetero- or homozygous for the Cia2:: F[1,2]; Mms19:: F[3] fusion proteins in diploid cells, and in haploids. **(C) Distribution of the scores following data analysis and filtering for the rPCA screen between Cia2:: F[1,2] and Mms19:: F[3].** The bright and dark gray bars represent the deletion (DMA) and temperature sensitive libraries, respectively. **(D) Functional annotations of genes identified in the rPCA screen.**

An initial indication of the feasibility of rPCA could be obtained by demonstrating that factors that alter the formation of a specific PPI would affect resistance to MTX. To test this, we selected a specific PPI from the yeast DHFR network (Cia2:: F[1,2]; Mms19:: F[3]) [5]; these two proteins have conserved roles in the biosynthesis and delivery of the iron-sulfur (Fe-S) cofactors [11], [12]. We then analyzed the effect of a *cis*-acting point mutation in Cia2 (*cia2-* E208G::F[3]), which we recently found (unpublished data) to impair its interaction with Mms19. The results show that while the Cia2-E208G mutant is stably expressed (Figure S2A), there is a clear difference in the resistance of the mutated cells to MTX, relative to the control, and indicate that the mDHFR activity can be eliminated by abrogating the protein-protein interaction (Figure 2A). Furthermore, in order to provide evidence demonstrating the specificity of the effect of this mutant on the interaction with Mms19, we dissected tetrads originating from a strain heterozygous for a knock out in the essential gene *CIA2* (*cia2*::*KmX*/*CIA2*), and harboring a *URA3* marked centromeric plasmid expressing the *CIA2-E208G* mutant. The results show that the *CIA2-E208G* mutant can support the growth of haploid spores knocked-out in the endogenous *CIA2* (Figure S2B), and suggests that the E208G replacement does not have a general effect on Cia2 function, and that the impaired interaction with Mms19 is likely to be specific. As a further control, we altered the highly reactive cysteine in Cia2 to alanine (C161A), which in contrast to the E208G allele was previously shown to cause lethality [17]. As shown in Figure 2A Cia2-C161A::F[3] construct still interacted with the MMS19::[F3] fusion protein, which supports the idea that E208G mutation is specific for Mms19.

Next, we asked whether our system provides a quantitative readout of the interaction levels of the proteins studied. We compared growth on MTX of haploids carrying the interacting pair Cia2:: F[1,2] and Mms19:: F[3] with isogenic strains hetero- or homozygous for the fusion proteins. While in the haploids and homozygous diploids, only the fusion proteins are available, in the heterozygous diploids, the untagged proteins (Cia2 and Mms19) can compete for binding with those that are tagged, and we therefore expected the level of reconstituted mDHFR to be reduced. Indeed, the results show a significant reduction in the ability to grow on MTX of the heterozygote, compared to the homozygous strain (Figure 2B, *left* panels). Furthermore, since the rPCA procedure is based on the evaluation of colony size, we repeated the same experiment, this time by recording colony sizes subsequent to robotic pinning. The expected fourfold reduction in reconstituted mDHFR in the heterozygous diploid strain was associated with similar fold reduction in colony size (Figure 2B, *right* panels).

Taken together, these results suggest that colony size on MTX containing media provides a quantitative readout indicating the changes in PPIs *in-vivo*. The range of the signal detectable in rPCA should depend on the quantity of complexes formed, which in turn is determined by the reduction in the abundance of the proteins studied, and their affinity for each other on various genetic backgrounds.

### Genome-wide rPCA screen: Data filtering and quality assessment

We next scaled up our procedures, to test the rPCA method described above for a total of four different screens. The logic behind our choice, to systematically identify mutants that dissociate the PPI between Cia2 and Mms19, was based on our long-term interest in pathways that play a role in genome stability. These proteins are part of a well-characterized and conserved complex that plays a key role in DNA metabolism, through a defined wide range of known and putative protein interactions (see below for more details). Furthermore, it was shown that a third member of this complex, Cia1, is able to bridge the complex, and therefore could serve as an important positive control [12], [18], [19].

The three other screens were mainly used to identify and eliminate potential sources of false positives (see below). In general, the query strains were screened at least two times against collections of ∼6000 mutants in both the non-essential and essential genes, which together cover almost the entire yeast genome. Screens were carried out in 1536 density format with four replicate copies of each mutant on the array, allowing four repeats for each screen. For the temperature sensitive mutants of essential genes, the experiments were performed at three different temperatures. The degree of interaction perturbation was quantified by calculating the ratio of colony size on medium with and without MTX. We measured the expected variability in colony size by calculating the standard deviation in the Cia2 and Mms19 screen, for each of the genes, based on the four replicate copies of each mutant on the array (Table S3). The standard deviation of temperature sensitive mutants was calculated for the optimal temperature selected for each particular gene (for more details see Materials and Methods, under the “Genome-wide rPCA screen, data filtering”). The average standard deviation was estimated at 0.0425.

The results obtained following data filtering (see Materials and Methods for more details) are presented in Figure 2C and 2D. We chose a threshold of 0.1, which represents ∼1% of the assayed genes: All mutants below this threshold are considered to affect the studied PPI. Results were analyzed using the Gene Ontology (GO) Term Finder (http://db.yeastgenome.org) at the Saccharomyces Genome Database to look for terms enriched among this mutant set.

We also accounted for two potential sources of false positives in an rPCA screen: 1) mutants in genes that specifically participate in DHFR biogenesis, and thus impair growth on MTX medium even when the mDHFR fragments still associate, and 2) mutants that have general effects on the interacting proteins, such as genes that affect protein biosynthesis, trafficking, RNA processing etc., and are therefore expected to be identified as positive hits in most screens. To control for these factors, we first generated and screened a strain harboring a fused F[1,2] and F[3] mDHFR cassette. This cassette provides complete resistance to MTX independently of any PPI. Next, we performed additional rPCA screens using PPIs unrelated to the Fe-S cluster biogenesis pathway. These screens included PPIs between the 26S proteasome subunits Rpn5::F[1,2] and Rpn11::F[3], and the pair of histone Htb2::F[1,2] and nucleosome remodeling protein, Nhp6a::F[1,2] (the complete results obtained from these screens are provided in Tables S4, S5, S6).

To ensure robustness of the false positive selection, we used a threshold of 0.2 for Cia2-Mms19, Htb2-Nhp6a, and Rpn5-Rpn11 screens. For the mDHFR cassette screen, a threshold of 0.25 was chosen, since the mDHFR strains are significantly less sensitive to MTX. For example, *APN1*, a classic expected false positive (see below), had a score 0.09 in the Cia2-Mms19 screen and 0.24 in the mDHFR screen. Genes that were considered false positives were those that passed the defined threshold in all three of the screens. As expected, in addition to *APN1*, we also identified *FOL1*; both were previously shown to play a role in the folic acid biosynthesis pathway, and therefore impaired DHFR biogenesis [20], [21]. Moreover, these screens also allowed us to eliminate 27 additional mutants that passed the threshold, and therefore were considered non-specific (shown in Table S7).

Alongside genes that were categorized as non-specific in the screen with Htb2::F[1,2] and Nhp6a::F[1,2], we were also able to identify genes that play a specific role in chromatin biology, and were previously shown to have genetic or physical interactions with Htb2 and Nhp6a (Table S1). One example is Htz1, histone variant H2AZ, that is exchanged for histone *H2A* in nucleosomes by the SWR1 complex. This protein was previously identified as a physical interactor of Htb2 and Nhp6a [22]–[24]. Furthermore, we also found Swr1, the catalytic subunit of the SWR1 complex and the main scaffold for the assembly of the complex, among the hits from this screen. This protein also physically interacts with Htb2 [24]. When extending the threshold to 0.2, we could identify several more candidates with biological significance. For example, Vps71 and Vps72 are two additional subunits of the SWR1 complex; Rsc8, Rsc9, Swi6 and Arp7 are components of two additional remodeling complexes, RSC and SWI/SNF, which show a synthetic growth defect with Nhp6a [25], [26], and also facilitate the binding of Nhp6a to nucleosomes [26]. This may be consistent with the fact that temperature sensitive alleles of RSC show a perturbation of the Nhp6a-Htb2 interaction.

In contrast to the screen with Htb2 and Nhp6a, in the case of Rpn5::F[1,2]-Rpn11::F[3], we could not detect meaningful biological connections (Table S5). Apparently, there are cases in which the PPIs are direct, without the need of any modifications. In such screens, we expect to identify genes that affect protein biogenesis in general and not hits with direct relevance to the interacting protein pair. Furthermore, cells can show robustness to the loss of some protein complexes, while being highly sensitive to the loss of others [27], [28]. Rpn5 and Rpn11, two essential proteasomal lid subunits, might represent one end of this spectrum. Essential protein complexes such as the proteasome are robust to genetic perturbations because the deletion of a subunit can be buffered by the modification of PPIs by other subunits, particularly by paralogous proteins. This functional compensation would likely lead to a relatively stable and functional alternative configuration. This notion is supported by previous studies showing that essential protein complexes consist of redundant subunits that render them robust to genetic perturbations [27], [28], and others showing that abundance of one paralogous protein is increased in response to the deletion of the other, at the levels of both transcript and protein abundances [29], [30].

### The rPCA screen enables systematic identification of mutants that mediate PPI between Cia2:: F[1,2] and Mms19:: F[3]

As mentioned above, Cia2 and Mms19 are proteins with conserved roles in the biosynthesis and delivery of the iron-sulfur (Fe-S) cofactors. Fe-S clusters are small inorganic cofactors found in hundreds of proteins that are required in virtually all phyla of life from bacteria to humans, and serve in electron transfer, enzyme catalysis, regulation of gene expression, and stabilization of protein structures. The biosynthesis of cellular Fe-S clusters is a complex process, starting at the mitochondria, which harbor the iron-sulfur cluster (ISC) assembly machinery. Mitochondria contribute to the maturation of cytosolic and nuclear Fe-S proteins as they export to the cytosol a still unidentified, sulfur-containing component which is used to assemble a Fe/S cluster on scaffold proteins. Cia2 and Mms19 form the part of the conserved cytoplasmic iron-sulfur protein assembly (CIA) machinery that specifically transfers and inserts the Fe-S cluster into target apoproteins (for review [31]).

**Figure 3:**
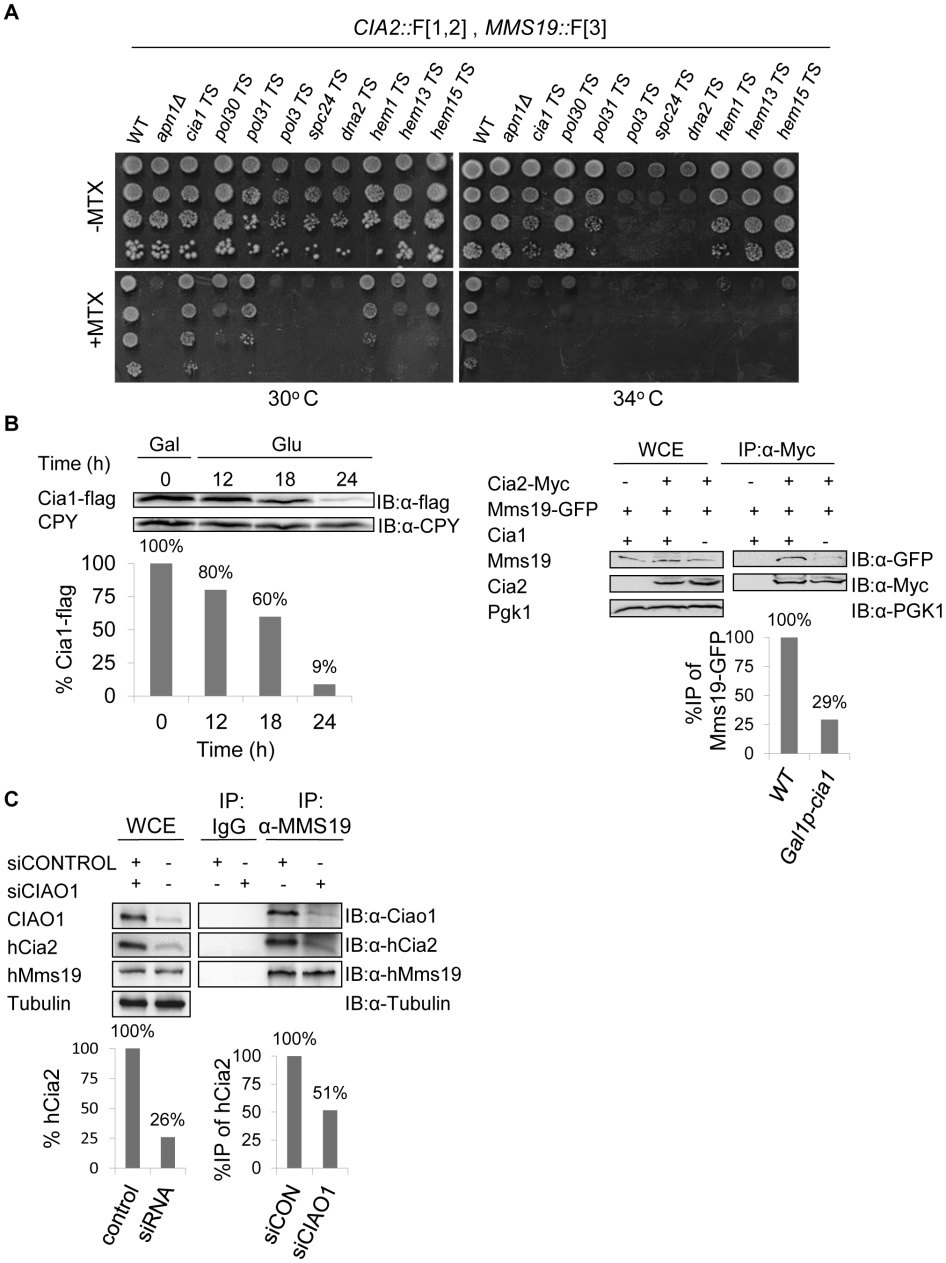
(A) Retesting of mutants for impaired growth on MTX-containing medium. Selected candidates from a rPCA screen against a query strain harboring the fusion proteins Cia2:: F[1,2] and Mms19:: F[3], were backcrossed to a wild type strain, sporulated, and subjected to tetrad dissection. Ten-fold serial dilutions of the indicated haploid spores were spotted on a rich medium lacking (control), or supplemented with 200 µg/ml MTX. Cells were incubated at 30°C, 34°C, and 37°C to identify the semi-permissive temperature of each temperature sensitive (Ts) mutant (30°C and 34°C are shown). A wild-type strain was used as a positive control. Δ*apn1*, which plays a specific role in DHFR biogenesis, was used as a negative control for growth on MTX. **(B) Reduced association between Mms19 and Cia2 in yeast cells depleted for Cia1, a member of the CIA complex.** *Left*-Western blot confirming that the expression levels of Cia1 regulated by a galactose-inducible promoter (*GAL1*), are abolished in glucose containing media. The expression of *GAL1*-*CIA1*-FLAG was induced by growing the cells in 2% galactose (Gal) for 3 hours (t-0). Then, 2% glucose (Glu) was added to the media to shut-off the expression of *CIA1*, and samples were collected at the indicated time points for immunoblotting with anti-FLAG, and anti carboxypeptidase-Y (CPY) ([IB]:α-CPY) (loading control). Quantitation of the band representing the Cia1-FLAG relative to the CPY loading control is shown in the graph. *Right*- Protein extracts were prepared from yeast strains carrying a galactose-inducible *CIA1*, and the combinations of Cia2-Myc and Mms19-GFP grown in 2% glucose for 24 hours (to deplete the levels of Cia1). After immunoprecipitation (IP) with anti-myc antibody, whole cell extracts (WCE), and immunocomplexes (IP:α-Myc) were separated by SDS-PAGE, and immunoblotted with anti-GFP and anti-Myc antibodies. Immunoblotting with anti-phosphoglycerate kinase1 (PGK1) antibody was used as a loading control. A strain carrying Mms19-GFP served as a negative control. Quantitation of the bands representing the precipitated Mms19-GFP relative to the total amount of Mms19 (WCE) is shown in the graph. **(C) siRNA-mediated depletion of CIAO1 in HeLa cells affected the endogenous levels of hCia2, and its association with hMms19.** Protein extracts were prepared from HeLa cells following the siRNA mediated knock down of CIAO1 (siCIAO1), or a non-targeting siRNA control (siCONTROL). WCE and immunoprecipitates (IP:α-Mms19) were separated by SDS-PAGE. hCia2, hMms19 and CIAO1 were immunostained using their specific antibodies. α-tubulin was used as a loading control. Quantitation of the band representing the hCia2 in the WCE relative to tubulin loading control is shown in the *left* graph. Quantitation of the bands representing the precipitated hCia2 relative to the amount of hCia2 in the WCE, and immunoprecipitated Mms19 is shown in *right* graph.

The rPCA screen between Cia2::F[1,2] and Mms19::F[3] identified 56 mutants with scores below the threshold value (Figure 2D and Table S2). The 56 top hits from the primary screen were reconfirmed by re-arraying on the control plates in 16 replicate copies, and then pinning on the MTX containing media (Figure S2C). We retested the candidates that were selected for further analysis by backcrossing to a wild type strain. Following meiosis and tetrad dissection, we confirmed that (1) the sensitivity to MTX segregates in a Mendelian manner (2∶2), indicating that it depends on a single gene mutation; (2) MTX sensitivity is linked to *URA3*, or *kanMX* (which were introduced as markers for the Ts alleles and the deletion mutants respectively), and therefore cosegregates with the mutated gene. Indeed, the selected spores that resulted from the backcross, show clear sensitivity to MTX at the semi-permissive temperatures (Figure 3A).

### Reduced association between Mms19 and Cia2 as a result of a mutation in Cia1, a member of the CIA complex

Based on Gene Ontology (GO) Term Finder annotations, we found functional groups that are likely to play a general role in protein synthesis and maturation, such as transcription and RNA processing, or protein degradation. Based on the rationale for selecting the list of false positives (see above), some of these genes passed the threshold in less than three of the screens, and therefore were retained as candidates that specifically impair the interaction between Mms19 and Cia2. The genes represented in our list of false positives probably play a general role in protein biosynthesis and modification. Nevertheless, we speculate that the genes that can be categorized as “general findings” in each of our screens, and were not included in our list of false positives, probably play a specific role in the biological pathways that were tested in this study. Moreover, although it was recently shown that the CIA complex mediates the transfer and insertion of the Fe-S cluster into target apoproteins that play roles in DNA metabolism and genome stability (see below), Fe-S clusters are also found in target proteins that participate in other fundamental biological processes such as ribosome biogenesis, enzyme catalysis, regulation of gene expression, stabilization of protein structures, etc. Although we believe that most of the genes are general findings (see above), we cannot rule out the possibility that some are specifically related to iron-sulfur biology.

In addition to the general functional groups, *CIA1* was one of the top hits which affected the interaction between Cia2::F[1,2] and Mms19::F[3], and served as an important quality control. Data from previous studies revealed that the CIA protein, Mms19, forms a complex with other late-acting CIA subunits both in yeast and human cells, including Cia1 (hCiao1), and Cia2 (hCia2; also termed *FAM96B*) [11], [12], [18]. Hence, we expected to find that a component of the CIA complex would affect its assembly. We further confirmed this result biochemically by co-IP experiments in yeast and mammalian cells. The potential role of Cia1 in mediating the interaction between Cia2 and Mms19 was examined by depleting the protein in a galactose- regulatable *CIA1*-FLAG strain, followed by IP. The expression of *CIA1*-FLAG was induced by growing the cells in galactose (Gal). Then glucose (Glu) was added to the media to shut-off the expression, and samples were collected at the indicated time points for immunoblotting with anti-FLAG. The results (Figure 3B-*left*) show that the expression levels of Cia1 were reduced by 91% after 24 hours. An IP experiment on the sample depleted in Cia1 (Figure 3B-*right*), shows that the depletion of *CIA1* did not affect the endogenous levels of Mms19 and Cia2, however, its depletion reduced the association of Mms19 with Cia2. This may suggest that in yeast, Cia1 is an adaptor protein for Cia2 and Mms19.

In support of this function, we obtained similar results in a strain harboring Cia2 and Mms19 fused to the Myc and TAP tags, respectively (Cia-Myc, and Mms19-TAP), and the original Ts allele of *CIA1*, grown for 24 hrs at the semi-permissive temperature (*cia1*-Ts). We also FLAG-tagged this allele at the C-terminus, and show that similarly to *GAL1-CIA1*, under these conditions expression of Cia1-Ts protein is abolished (Figure S3A).

Furthermore siRNA-mediated depletion of *CIAO1* in HeLa cells, led to almost four-fold decrease in the endogenous levels of hCia2 (Figure 3C-*left*). Moreover, only 50% of the available hCia2 co-IPed with hMms19, in the experimental sample (siCIAO1) (Figure 3C-*right*). These results suggest that in human cells, Ciao1 is a scaffold protein that also stabilizes hMms19 and/or hCia2. Collectively these data suggest that Cia1, like other members of the CIA complex, serves as a molecular scaffold that is required for its proper activity.

Another quality control was the discovery that the interaction between Cia2::F[1,2] and Mms19::F[3], is also affected by mutated genes that are involved in DNA metabolism, including components of the DNA replication and repair machinery, or those mediating mitotic chromosome segregation (Figure 2D and Table S2). Very recent studies have characterized Fe-S protein biogenesis as a key pathway for the maintenance of genomic integrity [11], [12]. Studies in both yeast and human cells revealed that cytoplasmic Mms19 binds to multiple nuclear Fe-S proteins involved in DNA metabolism [12], and suggested that the CIA complex targets Fe-S clusters to Fe-S apoproteins that are involved in genome stability. Indeed, components of the replication machinery, such as Pol2, Pol3, Pol31, and Dna2, that were shown to play an important role in genome integrity [32], were shown to require Fe-S clusters for their complex formation and activity [33], [34]. Our screen demonstrated that mutants in these specific Fe-S apoproteins (Table S2) perturb the PPI between Cia2 and Mms19. This may indicate that, similarly to other catalytic complexes such as the splicesosome [35], assembly of the CIA complex occurs on its substrates. In order to provide further support for this possibility, we decided to confirm biochemically by co-IP experiments the results showing that the interaction between Cia2::F[1,2] and Mms19::F[3] is affected by the temperature sensitive alleles of *DNA2*, and *POL3*, two previously described Fe-S targets [33], [34], and by *SPC24*, a protein involved in chromosome stability through its role in kinetochore clustering [36]. The potential role of these proteins in mediating the interaction between Cia2-Myc and Mms19-TAP was examined by growing the cells at the semi-restrictive temperature, followed by IP. The results show that while in all cases, Mms19 and Cia2 were stable, we could identify reduced association of Mms19 with Cia2 (Figure S3B). The question that arises from this result is why depletion of a single substrate would affect the interaction, since there are many other substrates still present in the cell. While revising our manuscript, we became aware of a very recent manuscript by R. Lill and colleagues [37]. In this study, the human *CIA2A* (*FAM96A*)-*CIA1* was identified as a component of the CIA machinery, together with *CIA2B* (*FAM96B*)-*CIA1*-*MMS19*, which was used in our study. Importantly they show that *CIA2B-CIA1-MMS19* specifically binds to and facilitates assembly of Fe-S targets involved in DNA metabolism and protein translation, while *CIA2A-CIA1* assists different branches of Fe-S protein assembly. This result suggests that the pool of Fe-S proteins targeted by the *CIA2B-CIA1-MMS19* complex is limited to specific functions. Thus, it is expected that specific mutations in a major pathway of the *CIA2B-CIA1-MMS19* machinery (such as the replisome) would significantly reduce the levels of reconstituted mDHFR in the cells.

The impressive list of proteins that were found to be associated with the CIA complex, and the enrichment of genome stability proteins [12], suggests that numerous other Fe-S cluster containing proteins are required for the maintenance of nuclear genome integrity. It is currently unclear which of these interactions are physiologically meaningful, as their identification and functional characterization are still pending. The identification of known Fe-S proteins in our rPCA screen suggests that the additional proteins identified may represent novel Fe-S cluster-containing proteins, or proteins that play different roles in the assembly of these apoproteins into stable complexes that can be detected by the CIA machinery.

### The stability of the CIA complex is regulated by iron levels

**Figure 4:**
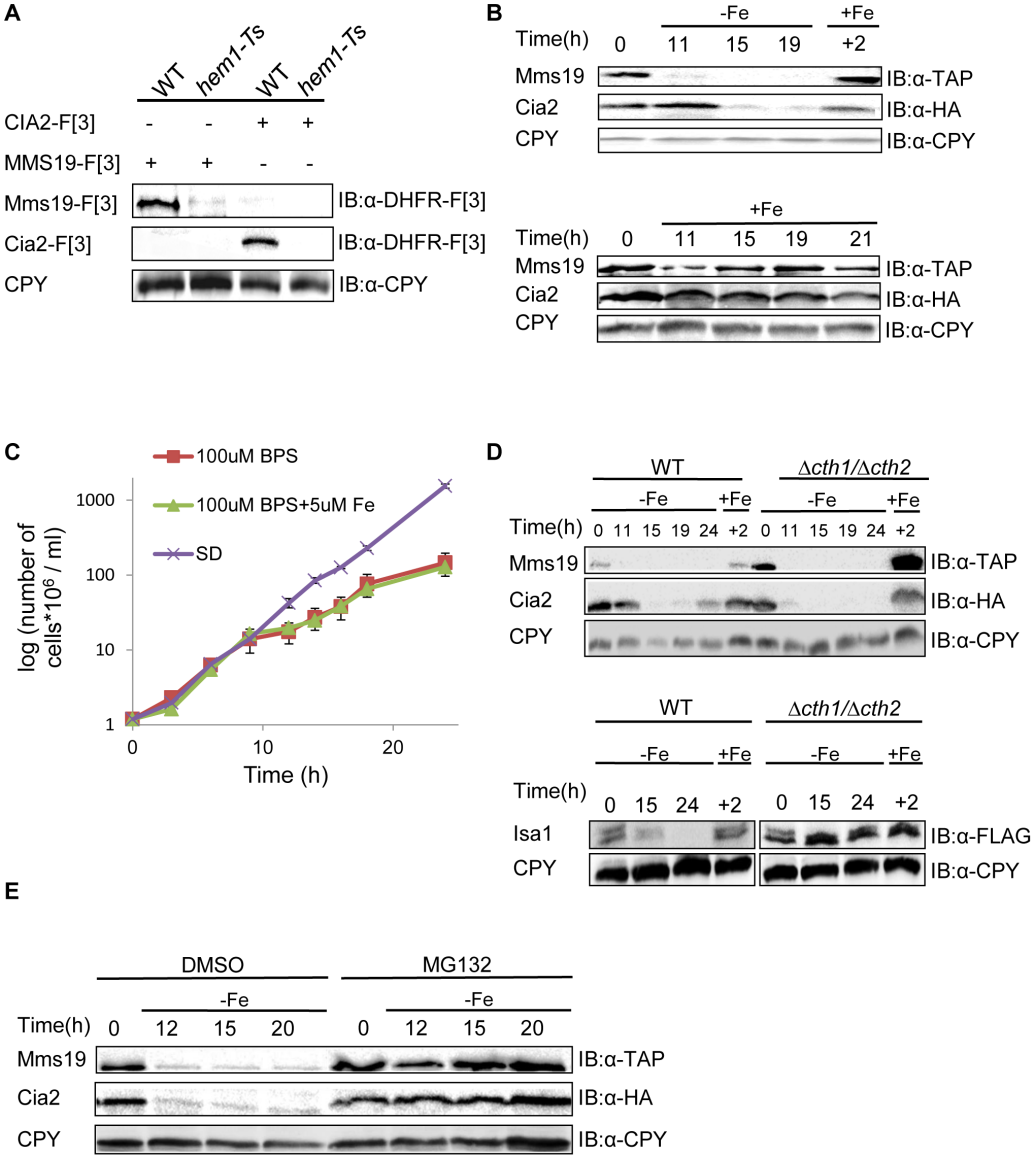
(A) The temperature sensitive allele of *HEM1* leads to a significant decrease in the endogenous levels of Cia2 and Mms19. Protein extracts were prepared from yeast strains carrying combinations of Mms19-F[3], Cia-[F3], the Ts allele of *HEM1*, and the wt control. Cell extracts were separated by SDS-PAGE, and immunoblotted with α-DHFR-F[3] antibody, and α-carboxypeptidase Y (CPY), which was used as a loading control. **(B) Reduced expression of Mms19 and Cia2 following -Fe depletion.** *Top*, wt cells in logarithmic growth expressing Mms19-TAP and Cia2-HA (t-0), were washed and re-suspended under Fe-limiting conditions (−Fe) (rich medium containing 100 µM of the Fe-chelator BPS, supplemented with 5 µM Fe), and then transferred back to rich medium (+Fe) for 2 hours (+2). Samples were collected at the indicated time points, and cell extracts were used for western blot analysis, and immunoblotting with α-TAP, α-HA, and α-CPY. *Bottom*, control experiment in which cells were grown in rich medium (+Fe) throughout the experiment. **(C) Growth curves of the wt strain grown under −Fe limiting conditions.** Mid-logarithmic wt cells were washed and re-suspended in rich medium (SD), −Fe limiting conditions were as used in the experiment described in B (100 µM BPS+5 µM Fe), with medium lacking Fe (100 µM BPS). O.D_600_ was measured at the indicated time points. **(D) The degradation of Mms19 and Cia2 upon Fe-depletion is independent of** ***CTH1*** **and** ***CTH2*** **.** The experimental design was similar to that described in B. This time, the degradation of Mms19 and Cia2 in wt cells was compared to cells deleted in *CTH1* and *CTH2*. *Bottom*, The strain containing a C-terminal FLAG tagged *ISA1* was used as a positive control for the deletions in *CTH1* and *CTH2*. **(E) The proteasome mediates the degradation of Mms19 and Cia2 upon Fe-depletion.** The experimental design was similar to that described in B. This time, the degradation of Mms19 and Cia2 was tested in the presence of DMSO (control), and in the presence of 25 mM of the proteasome inhibitor, MG132.

Our screen also identified *HEM1*, *HEM13*, and *HEM15*, genes involved in Heme biogenesis of, a prosthetic group that consists of an iron ion contained at the center of a large heterocyclic organic ring [38]. The assembly of both Fe-S clusters and Heme biogenesis are tightly regulated by iron homeostasis. We therefore further explored why heme mutants perturb the PPI between Cia2 and Mms19. As a first step, we attempted to confirm the result by co-IP. Surprisingly, we found that the dysfunctional allele of *HEM1* led to a significant decrease in the endogenous expression levels of both Cia2-F[3] and Mms19-[F3] (Figure 4A). Detailed genetic analysis from previous studies have shown that in yeast cells with reduced heme synthesis, the transcription of selected iron regulon genes is decreased, and leads to lower iron uptake into the cell [39], [40]. Given the finding that heme deficiency leads to reduced iron levels within the cells, we predicted that the endogenous levels of Cia2 and Mms19 might be regulated by iron availability. To test this, we assayed the expression of Cia2, and Mms19 fused to the HA and TAP tags, respectively (Cia-HA, and Mms19-TAP) at various time intervals under iron deprivation conditions. Cia-HA and Mms19-TAP were degraded in the presence of the Fe (II) specific chelator, bathophenantholine disulfonic acid (BPS) (Figure 4B). To rule out the possibility that this degradation was the result of cell death, we demonstrated that although their growth rate was delayed in the presence of BPS, cells were still in logarithmic growth at the indicated time points (Figure 4C); moreover, degradation of Cia2 and Mms19 was reversed by the addition of Fe (+Fe in Figure 4B).

Finally, we wished to provide further mechanistic insight into the degradation of Cia2 and Mms19 under Fe deficiency. Previous studies discovered a mechanism that mediates global posttranscriptional control of multiple components of Fe-dependent pathways to respond in a concerted fashion to Fe deficiency. In response to iron depletion, Aft1, the major iron regulon, induces the transcription of a specific set of genes involved in the activation of iron uptake, mobilization of intracellular stores of iron, and metabolic adaptation to iron limitation [41]. One of the activated genes, *CTH2*, coordinates this process by binding and targeting specific mRNA molecules to degradation [42]. We therefore used the approach described in Figure 4B, this time in cells deleted for *CTH2* (Figure 4D), or grown in the presence of the proteasome inhibitor MG132 (Figure 4E). Interestingly, in contrast to other proteins such as Isa1 which play a role in the early steps of Fe-S cluster assembly, and were shown to be degraded in a *CTH1/2* dependent manner (used as a control in Figure 4D-bottom), Cia2 and Mms19 were clearly stabilized as a result of proteasome inhibition, though not in Δ*cth1/2* cells (compare Fig. 4D, and 4E).

It is well established that iron deficiency in the mitochondria leads to decreased mitochondrial Fe-S protein biogenesis, at the mRNA level [42]. Our rPCA approach reveals for the first time that members of the CIA complex with essential roles at the final steps of Fe-S cluster biogenesis are part of the Fe-dependent pathways which are negatively regulated by Fe levels in a proteasome-dependent manner. This finding suggests that additional genes involved in cellular iron uptake should have been detected in our screen. We believe that since the screen was performed under ample iron availability, many of the genes regulated by Aft1 were not activated, and therefore, single deletions did not affect iron uptake, nor the protein levels of Mms19 and Cia2. Furthermore, the fact that almost none of these genes is essential [41], suggests that since the cell is not robust to reduced iron-uptake, redundancy in gene function allowed the cells to tolerate the loss of single genes in our screen. In this case hitting one gene may slightly induce another transporter to ensure proper iron uptake.

The PCA system represents an extremely powerful tool for the discovery of PPIs. The rPCA system extends this type of analysis to efficiently analyze trans-acting mutations that dissociate molecular interactions. We show that this technique is simple, and amenable to high throughput testing. Furthermore, PPI perturbations are detected in their endogenous environment in living cells and among proteins that are natively regulated. We validated this approach by demonstrating that previously characterized events leading to PPI dissociation can be reconstituted. Moreover, previously uncharacterized dissociation events could be specifically selected from large libraries using this genetic system. We believe that our study may lay the foundation for future comprehensive studies to study the effect of genetic perturbations on *in-vivo* PPI networks, and thus, is expected to promote further understanding of the eukaryotic interactome.

## Materials and Methods

### Yeast strains, media and growth conditions

All the strains used in this study are isogenic to BY4741, BY4742, or BY4743 [43]. The relevant genotypes are presented in Table S8. Myc, HA, and TAP fusions were generated using one step PCR mediated homologous recombination as previously described [44]. Strains from the PCA collection, containing different F[1,2], and F[3] fusion proteins were provided by Stephen W. Michnick's laboratory (University of Montreal). Galactose-regulatable *CIA1* strain was a gift from Roland Lill's laboratory (University of Marburg). The SGA markers were introduced into the query strains containing the fusion proteins of interest by crossing with Y7092 (*MAT* **a** *can1*Δ::*STE2pr-his5 lyp1*Δ *ura3*Δ0 *leu2*Δ0 *his3*Δ*1 met15*Δ0) [10]. Diploids were sporulated, and tetrads were dissected in order to select for the rPCA starting strain harboring the following drug resistances: X-F[1,2], Y-F[3] (clonNAT, and HygromycinB respectively), *can1*Δ::*STE2pr-his5* (canavanine) and *lyp1*Δ (thialysine).

*Saccharomyces cerevisiae* strains were grown at 30°C, unless specified otherwise. Standard YEP medium (1% yeast extract, 2% Bacto Peptone) supplemented with 2% galactose (YEPGal), or 2% dextrose (YEPD) was used for nonselective growth.

The media used in the rPCA analysis was a modification of the media used for SGA [10]. Drugs were added to the following final concentrations: canavanine (50 µg/ml, Sigma); thialysine (50 µg/ml, Sigma); clonNAT (100 µg/ml, Werner Bioagents); G418 (200 µg/ml, Invitrogen Life Technologies); methotrexate 200 µg/ml (prepared from a 10 mg/ml methotrexate in DMSO stock solution, Bioshop Canada); and HygromycinB (100 µg/ml, Calbiochem). Because ammonium sulfate impedes the function of G418 and clonNAT, synthetic medium containing these antibiotics was prepared with monosodium glutamic acid (MSG, Sigma) as a nitrogen source. Synthetic medium contained 0.1% Yeast nitrogen base w/o aa and Ammonium Sulfate, 0.1% Glutamic acid, 2% Dextrose, 0.2% amino acid mix, 4% noble agar (purified Agar, Bioshop).

The query strain were mated to the DMA on YEPD. Diploids were selected on YEPD supplemented with clonNAT and G418. Diploids were sporulated on a medium containing 2% agar and 10 gr/L potassium acetate. For selection of *MAT* **a** meiotic progeny carrying NatMX and, KanMX, and HygB markers, we used SD/MSG lacking histidine (to select for expression of *STE2pr-his5*), arginine, and lysine, and containing canavanine (to select for *can1*Δ), thialysine (to select for *lyp1*Δ), G418 (to select for KanMX), clonNAT (to select for NatMX), and HygromycinB (to select for HygB) [20 gr/L agar, 20 gr/L glucose, 1.7 gr/L yeast nitrogen base (SD/MSG – His/-Arg/-Lys+canavanine/+thialysine/+clonNAT/+G418/+HygromycinB)]. This medium was supplemented with methotrexate for the final selection step.

### Genome-wide rPCA screen, data filtering

Since the average ratio slightly varied from plate to plate, the obtained ratios from each plate were normalized to the common mean (0.4), which reflected the average impact of MTX on cell viability. Colony size on (−MTX) medium depends on the growth rate of the individual mutant strains. Data for mutants with colony size less than 70 pixels were eliminated, in order to remove dead and sick colonies, thereby avoiding experimental noise in data analysis.

For temperature sensitive mutants, the experiment was performed at three different temperatures, and the ratio was calculated for each temperature and normalized to the common mean, in a manner similar to the deletion mutants. Given that each temperature sensitive mutant has an optimal temperature for revealing its phenotype, we obtained the optimal ratio for each mutant by choosing the temperature providing the minimal ratio, while only a temperature resulting in a colony size greater than 70 pixels on −MTX media was considered. In order to estimate the variation in colony size, we calculated the standard deviation for each of the genes, based on the four replicate copies of each mutant on the array. The standard deviation of temperature sensitive mutants was calculated for the optimal temperature selected for the particular gene.

### Cell culture and siRNA transfection

HeLa cells were cultured in DMEM medium supplemented with 10% fetal bovine serum and 2 mM L-glutamine in a 37°C humidified incubator containing 5% CO2. siRNA duplexes targeting CIAO1 and non-targeting siRNA control were purchased from Dharmacon. Transient transfection of HeLa cells was performed using DharmaFECT 1 reagent as described by the manufacturer (Dharmacon).

### Mammalian whole-cell extract protein preparation and immunoprecipitations

HeLa cell pellets were lysed in RIPA buffer (20 mM Tris, pH 7.5, 150 mM NaCl, 1 mM EDTA, 1% Nonidet P-40, 0.5% sodium deoxycholate and 2 mM Na2VO4). For immunoprecipitations, lysates were incubated with anti-MMS19 (EUROMEDEX) antibody for 16 h at 4°C. Then 25 µl of protein A-G-agarose-conjugated beads were added, and the mixture was incubated for 1.5 h at 4°C. Beads were recovered by centrifugation, washed two times with TGET buffer (20 mM Tris HCl pH 7.5, 10% glycerol, 0.1% Triton X-100, 1 mM EDTA) supplemented with 150 mM NaCl and one time with TGET buffer supplemented with 75 mM NaCl. The beads were then boiled in SDS sample buffer for 5 min and briefly pelleted at 13,000 rpm before the supernatant was loaded for electrophoresis.

### Yeast whole-cell extract protein preparation and immunoprecipitations

A total of 5×10^8^ logarithmically growing cells were washed twice with water and resuspended in 1.5 ml of ice-chilled buffer B60 (50 mM HEPES-NaOH [pH 7.3], 0.1% Triton X-100, 20 mM β-glycerophosphate, 10% glycerol, protease inhibitor mix (Roche Biochemicals), 60 mM potassium acetate), and 1.5 g of ice-chilled glass beads was added. The tubes were vortexed eight times for 30 s with 30-s intervals on ice. After 10 min on ice, the lysate was decanted into ice-chilled 15-ml tubes and centrifuged for 20 min at 18,000×g at 4°C.

A 500-µl volume of clarified lysate was incubated with 25 µl of prewashed protein A-agarose beads at 4°C for 1 h. The beads were pelleted, and 450 µl of the lysate was transferred to a tube containing 7.5 µl of anti-myc antibody (Santa Cruz) and incubated at 4°C for 2 h. Then 25 µl of prewashed protein A-G-agarose-conjugated beads were added, and the mixture was incubated for 1 h at 4°C. The beads were then washed successively seven times: four times with B60 adjusted to 100 mM potassium acetate and once each with B60 adjusted to 210, 240, or 270 mM potassium acetate. The beads were boiled in SDS sample buffer for 5 min and briefly pelleted at 13,000 rpm in an Eppendorf centrifuge before the supernatant was loaded for electrophoresis.

### Western blots-data quantification

Image-J software was used to quantitate the western blots data. The protein level was calculated relatively to the loading control used in each experiment. In immunoprecipitation analysis the percentage of the precipitated protein was calculated relatively to the total protein level as indicated by the whole cell extract.

### Antibodies

Other antibodies used in this study were purchased commercially: anti-TAP (GenScript), anti-HA (Covance), anti-DHFR-[F3] (Sigma), anti-PGK1 (Molecular probes), anti-Ciao1 (Santa Cruz), anti-Cia2 Abcam), anti-alpha-Tubulin (Abcam), anti-CPY (Roche), anti-FLAG (Sigma).

## Supporting Information

Figure S1 **Changes in PPIs as a result of genetic perturbations could arise through several non-exclusive mechanisms.** In the following examples, deletion of gene C could lead (black arrow) to disruption of the A–B interaction through several mechanisms: (A) Protein C could represent a scaffold protein for protein A and B, or (B) stabilize protein A and/or B. (C) C could regulate the expression levels of A and/or B. (D) The A–B interaction could take place in a specific cellular compartment (such as the nucleus); in this example C represents a nuclear transporter, required for entry of protein A and/or B. (E) The A–B interaction could require a specific posttranslational modification such as phosphorylation on B (represented by P); in this example, C represents the protein kinase.
(TIF)

Figure S2 **The effect of** ***cia2-*** **E208G::F[3] mutant is specific to Mms19.** (A) The levels of Cia2- E208G::F[3], and Cia2-C161A::F[3] are relatively stable. Protein extracts were prepared from logarithmic yeast strains carrying *CIA2*::F[3] (control), *cia2-* E208G::F[3], and *cia2*-C161A::F[3]. Cell extracts were separated by SDS-PAGE and immunoblotted [IB] with the anti-DHFR-F[3] antibody ([IB]:α-DHFR-F[3]) and anti-CPY ([IB]:α-CPY) (loading control). (B) The *CIA2-E208G* mutant can support the growth of haploid spores knocked-out in the endogenous *CIA2*. Representative images of tetrad dissections originating from diploid strains heterozygous for deletion in *CIA2* (*cia2*::*KmX*). These strains were transformed with a *URA3* marked centromeric pRS316 plasmid (control), and the same plasmid expressing the WT *CIA2 (middle)*, or *CIA2-E208G* mutant (*right*). Strains were sporulated, and tetrads were dissected on rich media (YPD). By replica plating on G418 containing media, we confirmed that spores harboring the deletion in *CIA2* (white arrows) are lethal in the control. Replica plating on SD-Ura plate demonstrates that the presence of the centromeric plasmids (yellow arrows) can support growth only when expressing the wt *CIA2*, or *CIA2-E208G*. (C) Reconfirmation of the top hits that resulted from the rPCA screen between Cia2-F[3] and Mms19-[F3]. The 56 top hits from the primary screen were reconfirmed by re-arraying on the control plates in 16 replicate copies, and then pinning on the MTX containing media. Cells were incubated at 30°C, 34°C, and 37°C to identify the semi-permissive temperature of each of the Ts mutants (34°C and 37°C are shown). A wild-type strain was used as a positive control (Cont). *apn1*, and *fol1* mutants were used as a negative controls for growth on MTX (colored in red). These controls were randomly distributed on the plate. The candidates that were selected for further analysis are colored in yellow.
(TIF)

Figure S3 **Reduced association between Mms19 and Cia2 in yeast cells harboring the temperature sensitive (Ts) allele of CIA1.** *Left*-Evidence that the expression level of the temperature sensitive allele of CIA1 (*cia1*-Ts) is greatly reduced in cells grown at the semi-permissive temperature. Yeast cells carrying the Ts allele of *CIA1* fused to FLAG (*cia1*-FLAG-Ts), and the combinations of Cia-Myc, and Mms19-TAP were grown for 24 hours at the semi-permissive temperature (35°C). Samples were collected at the indicated time points for immunoblotting with anti-FLAG, and anti-CPY ([IB]:α-CPY) (loading control). Quantitation of the band representing the Cia1-FLAG-Ts relative to the loading control is shown in the graph. *Right*-The protein extracts from the same cells grown for 24 hours at 35°C were subjected to immunoprecipitation (IP) with anti-myc antibody; whole cell extracts (WCE) and immunocomplexes (IP:α-Myc) were separated by SDS-PAGE, and immunoblotted with anti-TAP, anti-Myc, and anti-CPY antibodies. Quantitation of the bands representing the precipitated Mms19-GFP relative to the total amount of Mms19 (WCE) is shown in the graph. (B) Reduced association between Mms19 and Cia2 in yeast cells harboring the temperature sensitive alleles of *DNA2* and *POL3*, two previously described Fe-S targets, and of *SPC24*, a protein involved in kinetochore clustering. Yeast cells carrying the Ts alleles of the indicated genes, and the combinations of Cia-Myc, and Mms19-TAP were grown for 24 hours at the semi-permissive temperature (35°C). Samples were subjected to immunoprecipitation (IP) with anti-myc antibody, and whole cell extracts (WCE) and immunocomplexes (IP:α-Myc) were separated by SDS-PAGE, and immunoblotted with anti-TAP or anti-Myc antibodies. The graph represents the quantitation of the precipitated Mms19-TAP relative to MMS19 in the corresponding sample in the WCE.
(TIF)

Table S1 **Genes Identified through Mutants that Affect the Physical Interaction Between** ***NHP6A*** **::F[1,2];** ***HTB2*** **::F[3].**
(PDF)

Table S2 **Genes Identified through Mutants that Affect the Physical Interaction Between** ***Cia2*** **::F[1,2];** ***Mms19*** **::F[3].**
(PDF)

Table S3 ***CIA2-MET18*** **screen results including the full data and the calculations used to achieve the final scores.**
(XLSX)

Table S4 **Fused** ***DHFR*** **screen results including the full data and the calculations used to achieve the final scores.**
(XLSX)

Table S5 ***RPN5-RPN11*** **screen results including the full data and the calculations used to achieve the final scores.**
(XLSX)

Table S6 ***HTB2-NHP6A*** **screen results including the full data and the calculations used to achieve the final scores.**
(XLSX)

Table S7 **List of false positives.**
(XLSX)

Table S8 **Yeast strains used in this study.**
(PDF)
