# Supplementary figures and images for: Reverse PCA, a Systematic Approach for Identifying Genes Important for the Physical Interaction between Protein Pairs
Source: PLoS Genet. 2013 Oct 10;9(10):e1003838. doi: 10.1371/journal.pgen.1003838 (PMC3794912; doi:10.1371/journal.pgen.1003838)

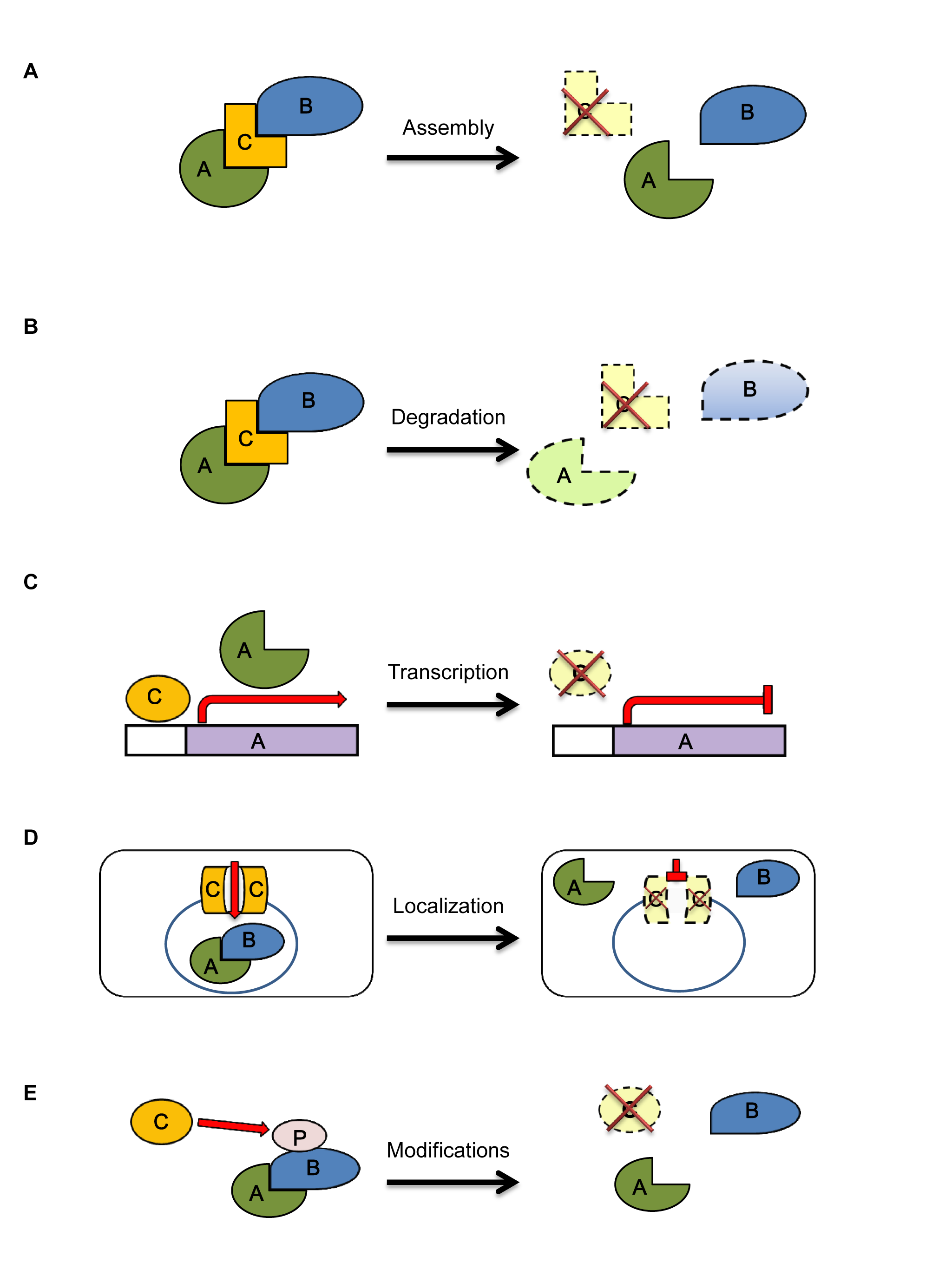

Supplement: Figure S1 — Changes in PPIs as a result of genetic perturbations could arise through several non-exclusive mechanisms. In the following examples, deletion of gene C could lead (black arrow) to disruption of the A–B interaction through several mechanisms: (A) Protein C could represent a scaffold protein for protein A and B, or (B) stabilize protein A and/or B. (C) C could regulate the expression levels of A and/or B. (D) The A–B interaction could take place in a specific cellular compartment (such as the nucleus); in this example C represents a nuclear transporter, required for entry of protein A and/or B. (E) The A–B interaction could require a specific posttranslational modification such as phosphorylation on B (represented by P); in this example, C represents the protein kinase. (TIF) [file pgen.1003838.s001.tif]

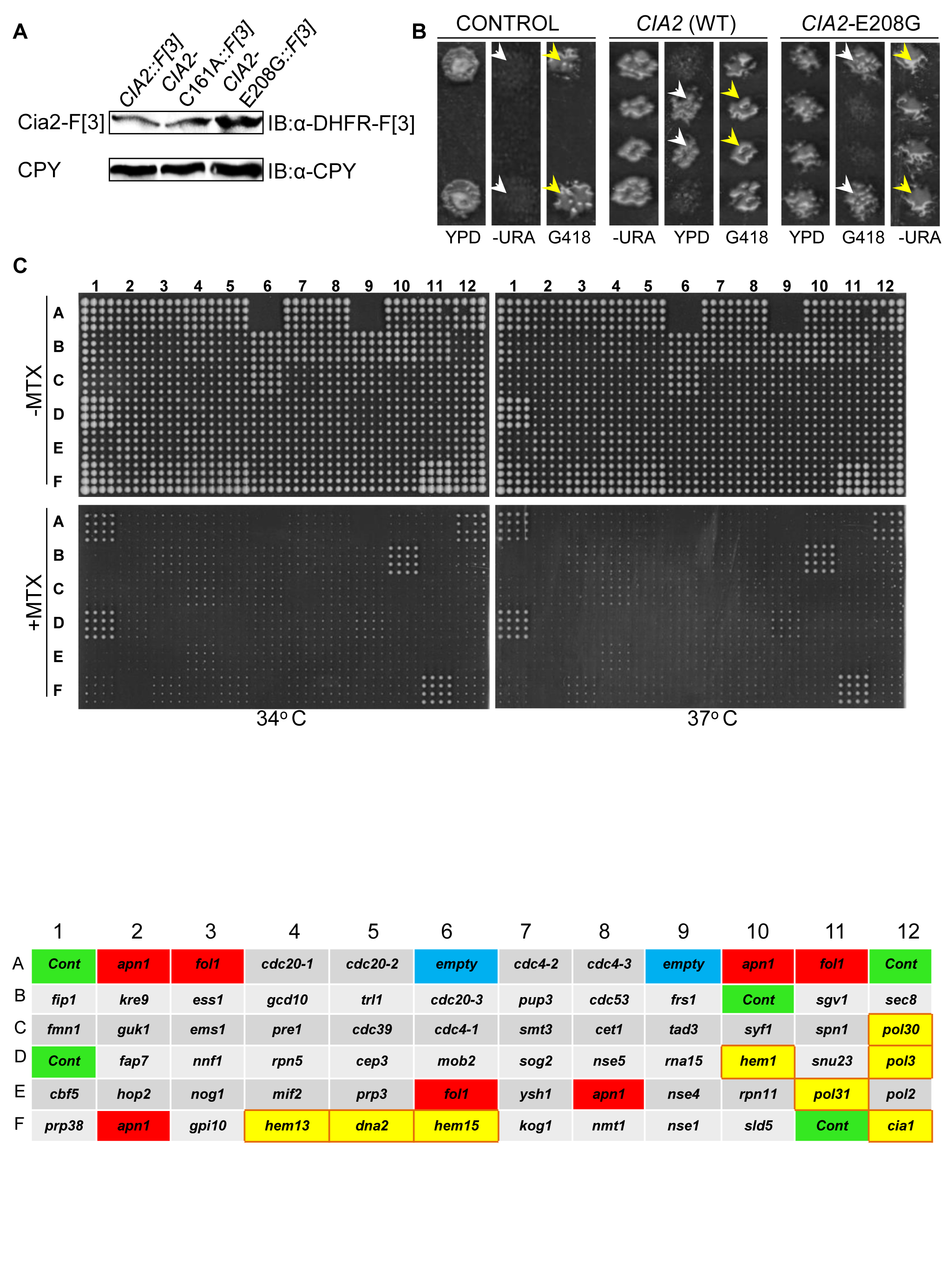

Supplement: Figure S2 — The effect of cia2- E208G::F[3] mutant is specific to Mms19. (A) The levels of Cia2- E208G::F[3], and Cia2-C161A::F[3] are relatively stable. Protein extracts were prepared from logarithmic yeast strains carrying CIA2::F[3] (control), cia2- E208G::F[3], and cia2-C161A::F[3]. Cell extracts were separated by SDS-PAGE and immunoblotted [IB] with the anti-DHFR-F[3] antibody ([IB]:α-DHFR-F[3]) and anti-CPY ([IB]:α-CPY) (loading control). (B) The CIA2-E208G mutant can support the growth of haploid spores knocked-out in the endogenous CIA2. Representative images of tetrad dissections originating from diploid strains heterozygous for deletion in CIA2 (cia2::KmX). These strains were transformed with a URA3 marked centromeric pRS316 plasmid (control), and the same plasmid expressing the WT CIA2 (middle), or CIA2-E208G mutant (right). Strains were sporulated, and tetrads were dissected on rich media (YPD). By replica plating on G418 containing media, we confirmed that spores harboring the deletion in CIA2 (white arrows) are lethal in the control. Replica plating on SD-Ura plate demonstrates that the presence of the centromeric plasmids (yellow arrows) can support growth only when expressing the wt CIA2, or CIA2-E208G. (C) Reconfirmation of the top hits that resulted from the rPCA screen between Cia2-F[3] and Mms19-[F3]. The 56 top hits from the primary screen were reconfirmed by re-arraying on the control plates in 16 replicate copies, and then pinning on the MTX containing media. Cells were incubated at 30°C, 34°C, and 37°C to identify the semi-permissive temperature of each of the Ts mutants (34°C and 37°C are shown). A wild-type strain was used as a positive control (Cont). apn1, and fol1 mutants were used as a negative controls for growth on MTX (colored in red). These controls were randomly distributed on the plate. The candidates that were selected for further analysis are colored in yellow. (TIF) [file pgen.1003838.s002.tif]

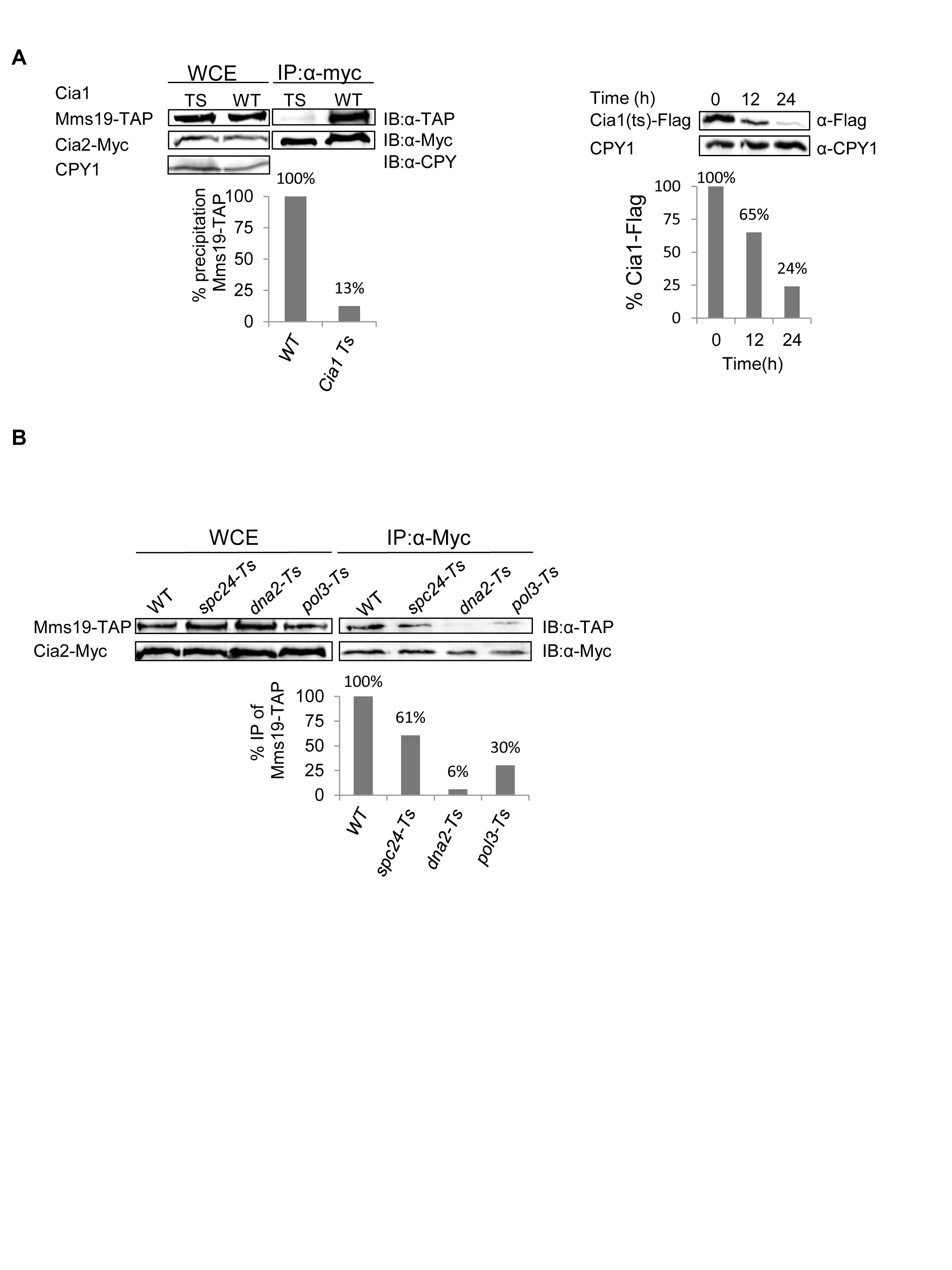

Supplement: Figure S3 — Reduced association between Mms19 and Cia2 in yeast cells harboring the temperature sensitive (Ts) allele of CIA1. Left-Evidence that the expression level of the temperature sensitive allele of CIA1 (cia1-Ts) is greatly reduced in cells grown at the semi-permissive temperature. Yeast cells carrying the Ts allele of CIA1 fused to FLAG (cia1-FLAG-Ts), and the combinations of Cia-Myc, and Mms19-TAP were grown for 24 hours at the semi-permissive temperature (35°C). Samples were collected at the indicated time points for immunoblotting with anti-FLAG, and anti-CPY ([IB]:α-CPY) (loading control). Quantitation of the band representing the Cia1-FLAG-Ts relative to the loading control is shown in the graph. Right-The protein extracts from the same cells grown for 24 hours at 35°C were subjected to immunoprecipitation (IP) with anti-myc antibody; whole cell extracts (WCE) and immunocomplexes (IP:α-Myc) were separated by SDS-PAGE, and immunoblotted with anti-TAP, anti-Myc, and anti-CPY antibodies. Quantitation of the bands representing the precipitated Mms19-GFP relative to the total amount of Mms19 (WCE) is shown in the graph. (B) Reduced association between Mms19 and Cia2 in yeast cells harboring the temperature sensitive alleles of DNA2 and POL3, two previously described Fe-S targets, and of SPC24, a protein involved in kinetochore clustering. Yeast cells carrying the Ts alleles of the indicated genes, and the combinations of Cia-Myc, and Mms19-TAP were grown for 24 hours at the semi-permissive temperature (35°C). Samples were subjected to immunoprecipitation (IP) with anti-myc antibody, and whole cell extracts (WCE) and immunocomplexes (IP:α-Myc) were separated by SDS-PAGE, and immunoblotted with anti-TAP or anti-Myc antibodies. The graph represents the quantitation of the precipitated Mms19-TAP relative to MMS19 in the corresponding sample in the WCE. (TIF) [file pgen.1003838.s003.tif]
