# Supplementary material for: Reverse PCA, a Systematic Approach for Identifying Genes Important for the Physical Interaction between Protein Pairs
Source: PLoS Genet. 2013 Oct 10;9(10):e1003838. doi: 10.1371/journal.pgen.1003838 (PMC3794912; doi:10.1371/journal.pgen.1003838)
Supplement: Table S1 — Genes Identified through Mutants that Affect the Physical Interaction Between NHP6A ::F[1,2]; HTB2 ::F[3]. (PDF) [file pgen.1003838.s004.pdf]

Table S1. Genes Identified through Mutants that Affect the Physical Interaction Between *NHP6A*::F[1,2]; *HTB2*::F[3]

Systematic Name	Standard Name	Description
**Chromatin remodeling**		
YOL012C	*HTZ1*	Histone variant H2AZ, exchanged for histone H2A in nucleosomes by the SWR1 complex; involved in transcriptional regulation through prevention of the spread of silent heterochromatin
YDR334W	*SWR1*	Swi2/Snf2-related ATPase that is the structural component of the SWR1 complex, which exchanges histone variant H2AZ (Htz1p) for chromatin-bound histone H2A
YGR270W	*YTA7*	Protein that localizes to chromatin and has a role in regulation of histone gene expression; has a bromodomain-like region that interacts with the N-terminal tail of histone H3, and an ATPase domain; potentially phosphorylated by Cdc28p
**Protein biosynthesis and modification**		
YDL132W	*CDC53*	Cullin, structural protein of SCF complexes (which also contain Skp1p, Cdc34p, Hrt1p and an F-box protein) involved in ubiquitination; SCF promotes the G1-S transition by targeting G1 cyclins and the Cln-CDK inhibitor Sic1p for degradation
YML092C	*PRE8*	Alpha 2 subunit of the 20S proteasome
YDR434W	*GPI17*	Transmembrane protein subunit of the glycosylphosphatidylinositol transamidase complex that adds GPIs to newly synthesized proteins
**Transcription and RNA processing**		
YGR006W	*PRP18*	Splicing factor involved in the positioning of the 3' splice site during the second catalytic step of splicing, part of snRNP U5, interacts with Slu7p
YPR178W	*PRP4*	Splicing factor, component of the U4/U6-U5 snRNP complex
YHR085W	*IPI1*	Essential component of the Rix1 complex (with Rix1p and Ipi3p) that is required for processing of ITS2 sequences from 35S pre-rRNA; Rix1 complex associates with Mdn1p in pre-60S ribosomal particles
YDL098C	*SNU23*	Component of U4/U6.U5 snRNP involved in mRNA splicing via spliceosome
YOR194C	*TOA1*	TFIIA large subunit; involved in transcriptional activation, acts as antirepressor or as coactivator; homologous to largest and second largest subunits of human and Drosophila TFIIA
**DNA replication**		
YOR060C	*SLD7*	Protein with a role in chromosomal DNA replication; interacts with Sld3p and reduces its affinity for Cdc45p; deletion mutant has aberrant mitochondria
**Transport and secretion**		
YOL130W	*ALR1*	Plasma membrane Mg(2+) transporter, expression and turnover are regulated by Mg(2+) concentration; overexpression confers increased tolerance to Al(3+) and Ga(3+) ions
YGR257C	*MTM1*	Mitochondrial protein of the mitochondrial carrier family, involved in activating mitochondrial Sod2p probably by facilitating insertion of an essential manganese cofactor
YDR246W	*TRS23*	One of 10 subunits of the transport protein particle (TRAPP) complex of the cis-Golgi which mediates vesicle docking and fusion; involved in endoplasmic reticulum (ER) to Golgi membrane traffic
YGR120C	*COG2*	Essential component of the conserved oligomeric Golgi complex (Cog1p through Cog8p), a cytosolic tethering complex that functions in protein trafficking to mediate fusion of transport vesicles to Golgi compartments
YDL145C	*COP1*	Alpha subunit of COPI vesicle coatomer complex, which surrounds transport vesicles in the early secretory pathway
YNL272C	*SEC2*	Guanyl-nucleotide exchange factor for the small G-protein Sec4p; essential for post-Golgi vesicle transport and for autophagy; associates with the exocyst, via exocyst subunit Sec15p, on secretory vesicles

YPR055W	*SEC8*	Essential 121 kDa subunit of the exocyst complex; the exocyst complex (Sec3p, Sec5p, Sec6p, Sec8p, Sec10p, Sec15p, Exo70p, and Exo84p) has the essential function of mediating polarized targeting of secretory vesicles to active sites of exocytosis; relocalizes away from bud neck upon DNA replication stress
YNR026C	*SEC12*	Guanine nucleotide exchange factor (GEF), activates Sar1p by catalyzing the exchange of GDP for GTP; required for the initiation of COPII vesicle formation in ER to Golgi transport; glycosylated integral membrane protein of the ER
YNL287W	*SEC21*	Gamma subunit of coatomer, a heptameric protein complex that together with Arf1p forms the COPI coat; involved in ER to Golgi transport of selective cargo
YGL137W	*SEC27*	Essential beta'-coat protein of the COPI coatomer, involved in ER-to-Golgi and Golgi-to-ER transport
YLR440C	*SEC39*	Component of the Dsl1p tethering complex that interacts with ER SNAREs Sec20p and Use1p; proposed to be involved in protein secretion; localizes to the ER and nuclear envelope
**Unknown**		
YMR134W	*ERG29*	Protein of unknown function that may be involved in iron metabolism; mutant bm-8 has a growth defect on iron-limited medium that is complemented by overexpression of Yfh1p; shows localization to the ER; highly conserved in ascomycetes

Biologically relevant proteins with a score between 0.1 and 0.2

**Chromatin remodeling**		
YML041C	*VPS71*	Nucleosome-binding component of the SWR1 complex, which exchanges histone variant H2AZ (Htz1p) for chromatin-bound histone H2A; required for vacuolar protein sorting
YDR485C	*VPS72*	Htz1p-binding component of the SWR1 complex; exchanges histone variant H2AZ (Htz1p) for chromatin-bound histone H2A
YFR037C	*RSC8*	Component of the RSC chromatin remodeling complex; essential for viability and mitotic growth; homolog of SWI/SNF subunit Swi3p
YML127W	*RSC9*	Component of the RSC chromatin remodeling complex; DNA-binding protein involved in the synthesis of rRNA and in transcriptional repression and activation of genes regulated by the Target of Rapamycin (TOR) pathway
YLR182W	*SWI6*	Transcription cofactor; forms complexes with Swi4p and Mbp1p to regulate transcription at the G1/S transition; involved in meiotic gene expression
YPR034W	*ARP7*	Component of both the SWI/SNF and RSC chromatin remodeling complexes; actin-related protein involved in transcriptional regulation
**Transcription and RNA processing**		
YBR193C	*MED8*	Subunit of the RNA polymerase II mediator complex; associates with core polymerase subunits to form the RNA polymerase II holoenzyme; essential for transcriptional regulation
YER022W	*SRB4*	Subunit of the RNA polymerase II mediator complex; associates with core polymerase subunits to form the RNA polymerase II holoenzyme; essential for transcriptional regulation
