# Supplementary material for: Reverse PCA, a Systematic Approach for Identifying Genes Important for the Physical Interaction between Protein Pairs
Source: PLoS Genet. 2013 Oct 10;9(10):e1003838. doi: 10.1371/journal.pgen.1003838 (PMC3794912; doi:10.1371/journal.pgen.1003838)
Supplement: Table S2 — Genes Identified through Mutants that Affect the Physical Interaction Between Cia2 ::F[1,2]; Mms19 ::F[3]. (PDF) [file pgen.1003838.s005.pdf]

Table S2. Genes Identified through Mutants that Affect the Physical Interaction Between *Cia2*::F[1,2]; *Mms19*::F[3]

Systematic Name	Standard Name	Description
**Iron-sulfur and Heme biogenesis (regulated by iron homeostasis)**		
YDR232W	*HEM1*	5-aminolevulinate synthase, catalyzes the first step in the heme biosynthetic pathway
YDR044W	*HEM13*	Coproporphyrinogen III oxidase, an oxygen requiring enzyme that catalyzes the sixth step in the heme biosynthetic pathway
YOR176W	*HEM15*	Ferrochelatase, a mitochondrial inner membrane protein, catalyzes the insertion of ferrous iron into protoporphyrin IX, the eighth and final step in the heme biosynthetic pathway
YDR267C	*CIA1*	Component of cytosolic iron-sulfur protein assembly (CIA) machinery; acts at a late step of Fe-S cluster assembly
**DNA replication and repair**		
YNL262W	*POL2*	Catalytic subunit of DNA polymerase (II) epsilon, a chromosomal DNA replication polymerase that exhibits processivity and proofreading exonuclease activity; also involved in DNA synthesis during DNA repair
YDL102W	*POL3*	Catalytic subunit of DNA polymerase delta; required for chromosomal DNA replication during mitosis and meiosis, intragenic recombination, repair of double strand DNA breaks, and DNA replication during nucleotide excision repair (NER)
YBR088C	*POL30*	Proliferating cell nuclear antigen (PCNA), functions as the sliding clamp for DNA polymerase delta
YJR006W	*POL31*	Subunit of DNA polymerase delta (polymerase III); essential for cell viability; involved in DNA replication and DNA repair
YDR489W	*SLD5*	Subunit of the GINS complex (Sld5p, Psf1p, Psf2p, Psf3p), which is localized to DNA replication origins and implicated in assembly of the DNA replication machinery
YLR007W	*NSE1*	Component of the SMC5-SMC6 complex; this complex plays a key role in the removal of X-shaped DNA structures that arise between sister chromatids during DNA replication and repair
YDL105W	*NSE4*	Component of the SMC5-SMC6 complex; this complex plays a key role in the removal of X-shaped DNA structures that arise between sister chromatids during DNA replication and repair
YML023C	*NSE5*	Component of the SMC5-SMC6 complex; this complex plays a key role in the removal of X-shaped DNA structures that arise between sister chromatids during DNA replication and repair
YHR164C	*DNA2*	Tripartite DNA replication factor; has single-stranded DNA-dependent ATPase, ATP-dependent nuclease, and helicase activities; required for Okazaki fragment processing; involved in DNA repair; cell-cycle dependent localization; forms nuclear foci upon DNA replication stress
YGL033W	*HOP2*	Meiosis-specific protein that localizes to chromosomes, preventing synapsis between nonhomologous chromosomes and ensuring synapsis between homologs
**Mitosis and chromosome segregation**		
YGL116W	*CDC20*	Cell-cycle regulated activator of anaphase-promoting complex/cyclosome (APC/C)
YFL009W	*CDC4*	F-box protein required for G1/S and G2/M transition
YDL126C	*CDC48*	AAA ATPase involved in multiple processes; subunit of a polyubiquitin-selective segregase complex involved in ERAD, cell wall integrity during heat stress and mitotic spindle disassembly
YMR117C	*SPC24*	Component of the evolutionarily conserved kinetochore-associated Ndc80 complex involved in chromosome segregation, spindle checkpoint activity and kinetochore clustering

YDR510W	*SMT3*	Ubiquitin-like protein of the SUMO family, conjugated to lysine residues of target proteins; regulates chromatid cohesion, chromosome segregation, APC-mediated proteolysis, DNA replication and septin ring dynamics
YFL034C-B	*MOB2*	Activator of Cbk1p kinase; activation of Cbk1p facilitates the Ace2p-dependent daughter cell-specific transcription of genes involved in cell separation; similar to Mob1p
YOR353C	*SOG2*	Key component of the RAM signaling network, required for proper cell morphogenesis and cell separation after mitosis
YMR168C	*CEP3*	Essential kinetochore protein, component of the CBF3 complex that binds the CDEIII region of the centromere
YKL089W	*MIF2*	Protein required for structural integrity of elongating spindles
YJR112W	*NNF1*	Essential component of the MIND kinetochore complex, required for accurate chromosome segregation
**Protein biosynthesis and modification**		
YDR454C	*GUK1*	Guanylate kinase, converts GMP to GDP; required for growth and mannose outer chain elongation of cell wall N-linked glycoproteins
YDR236C	*FMN1*	Riboflavin kinase, produces riboflavin monophosphate (FMN); FMN is a necessary cofactor for many enzymes; predominantly localizes to the microsomal fraction and also found in the mitochondrial inner membrane
YJL174W	*KRE9*	Glycoprotein involved in cell wall beta-glucan assembly; null mutation leads to severe growth defects, aberrant multibudded morphology, and mating defects
YGL142C	*GPI10*	Integral membrane protein involved in GPI anchor synthesis
YHR186C	*KOG1*	Subunit of TORC1, a rapamycin-sensitive complex involved in growth control that contains Tor1p or Tor2p, Lst8p and Tco89p; contains four HEAT repeats and seven WD-40 repeats
YLR195C	*NMT1*	N-myristoyl transferase, catalyzes the cotranslational, covalent attachment of myristic acid to the N-terminal glycine residue of several proteins involved in cellular growth and signal transduction
YER012W	*PRE1*	Beta 4 subunit of the 20S proteasome; localizes to the nucleus throughout the cell cycle
YDL147W	*RPN5*	Subunit of the COP9 signalosome (CSN) and non-ATPase regulatory subunit of the 26S proteasome lid
YFR004W	*RPN11*	Metalloprotease subunit of the 19S regulatory particle of the 26S proteasome lid; couples the deubiquitination and degradation of proteasome substrates
YER094C	*PUP3*	Beta 3 subunit of the 20S proteasome involved in ubiquitin-dependent catabolism; human homolog is subunit C10
YDL132W	*CDC53*	Cullin, structural protein of SCF complexes involved in ubiquitination; SCF promotes the G1-S transition by targeting G1 cyclins and the Cln-CDK inhibitor Sic1p for degradation
**Transcription and RNA processing**		
YJR093C	*FIP1*	Subunit of cleavage polyadenylation factor (CPF), interacts directly with poly(A) polymerase (Pap1p) to regulate its activity
YPL228W	*CET1*	Beta (RNA 5'-triphosphatase) subunit of the mRNA capping enzyme, a heterodimer (the other subunit is CEG1, a guanylyltransferase) involved in adding the 5' cap to mRNA
YGL044C	*RNA15*	Component of the cleavage and polyadenylation factor I (CF I); CF 1,is involved in cleavage and polyadenylation of mRNA 3' ends

YDL098C	*SNU23*	Component of U4/U6.U5 snRNP involved in mRNA splicing via spliceosome
YDR416W	*SYF1*	Member of the NineTeen Complex (NTC) that contains Prp19p and stabilizes U6 snRNA in catalytic forms of the spliceosome containing U2, U5, and U6 snRNAs
YLR277C	*YSH1*	Putative endoribonuclease, subunit of the mRNA cleavage and polyadenylation specificity complex
YDR473C	*PRP3*	Splicing factor, component of the U4/U6-U5 snRNP complex
YGR075C	*PRP38*	Unique component of the U4/U6.U5 tri-snRNP particle, required for conformational changes which result in the catalytic activation of the spliceosome
YLR175W	*CBF5*	Pseudouridine synthase catalytic subunit of box H/ACA small nucleolar ribonucleoprotein particles (snoRNPs)
YDL166C	*FAP7*	Essential NTPase required for small ribosome subunit synthesis
YPL217C	*BMS1*	GTPase required for synthesis of 40S ribosomal subunits and for processing the 35S pre-rRNA
YPL093W	*NOG1*	Putative GTPase that is required for 60S ribosomal subunit biogenesis
YJR017C	*ESS1*	Peptidylprolyl-cis/trans-isomerase (PPIase),required for efficient termination of mRNA transcription and trimethylation of histone H3
YCR093W	*CDC39*	Component of the CCR4-NOT complex, which has multiple roles in regulating mRNA levels
YLR060W	*FRS1*	Beta subunit of cytoplasmic phenylalanyl-tRNA synthetase
YLR316C	*TAD3*	Subunit of tRNA-specific adenosine-34 deaminase, forms a heterodimer with Tad2p that converts adenosine to inosine at the wobble position of several tRNAs
YPR161C	*SGV1*	Cyclin (Bur2p)-dependent protein kinase that functions in transcriptional regulation
YJL087C	*TRL1*	tRNA ligase, required for tRNA splicing and for both splicing and translation of HAC1 mRNA in the UPR
YNL062C	*GCD10*	Subunit of tRNA (1-methyladenosine) methyltransferase with Gcd14p, required for the modification of the adenine at position 58 in tRNAs, especially tRNAi-Met
YPR133C	*SPN1*	Protein involved in RNA polymerase II transcription
**Transport and secretion**		
YPR055W	*SEC8*	Essential 121 kDa subunit of the exocyst complex with the essential function of mediating polarized targeting of secretory vesicles to active sites of exocytosis
