# Supplementary material for: Reverse PCA, a Systematic Approach for Identifying Genes Important for the Physical Interaction between Protein Pairs
Source: PLoS Genet. 2013 Oct 10;9(10):e1003838. doi: 10.1371/journal.pgen.1003838 (PMC3794912; doi:10.1371/journal.pgen.1003838)
Supplement: Table S8 — Yeast strains used in this study. (PDF) [file pgen.1003838.s011.pdf]

Table S8. Yeast strains used in this study

Strain	Genotype	Reference
BY4741	*Mat**a** /his3Δ/ leu2Δ/ met15Δ/ ura3Δ*	[2]
BY4742	*MATα/ ura3Δ0/ leu2Δ0/ his3Δ1/ lys2Δ0*	[2]
YSB109	*Matα/can1Δ::ste2pr-Sp_HIS5/ lyp1Δ/ his3Δ/ leu2Δ / LYS1/ ura3Δ/ met15Δ/ MMS19::mDHFR F[3]- HygB/ CIA2::mDHFR F[1,2]-natMX*	This Study
YSB775	*Matα/lyp1Δ/ his3Δ/ leu2Δ / LYS1 / ura3Δ/ met15Δ/ MMS19::DHFR F[3]- HygB CIA2::DHFR F[1,2]-natMX*	This Study
YSB189	*Mat**a**/ leu2Δ0/his3Δ1/LYS1/met15Δ0/MMS19::DHFR F[1,2]-natMX* + pRS316-*CIA2- F[3]-HygB*	This study
YSB190	*Mat**a**/leu2Δ0/his3Δ1/LYS1/met15Δ0/MMS19::DHFRF[1,2]-natMX*+pRS316-*CIA2*- E208G-*F[3]-HygB*	This Study
YSB193	*Mat**a**/leu2Δ0/his3Δ1/LYS1/met15Δ0/MMS19::DHFRF[1,2]-natMX*+pRS316-*CIA2*-E161- *F[3]-HygB*	This Study
YSB402	*Mat α/ can1Δ::ste2pr-Sp_HIS5/ lyp1Δ/ his3Δ/ leu2Δ / LYS1 / ura3Δ/ met15Δ/ RPN5::DHFR F[1,2]-natMX / RPN11::DHFR F[3]- HygB/*	This Study
YSB403	*Mat α/ lyp1Δ/ his3Δ/ leu2Δ /LYS1 /ura3Δ/met15Δ/ RPN5::DHFR F[1,2]-natMX /RPN11::mDHFR F[3]- HygB/*	This Study
YSB603	*Matα/ lyp1Δ/ his3Δ/ leu2Δ / LYS1 / ura3Δ/ met15Δ/ NHP6A::mDHFR F[1,2+3]- HygB/*	This Study
YSB604	*Matα/can1Δ::ste2pr-Sp_HIS5/lyp1Δ/his3Δ/leu2Δ/LYS1/ura3Δ/met15Δ/ NHP6A::mDHFR F[1,2+3]- HygB*	This Study
YSB457	*Matα/can1Δ::ste2pr-Sp_HIS5/lyp1Δ/his3Δ/leu2Δ/LYS1/ura3Δ/met15Δ/ NHP6A::mDHFR F[1,2]-natMX / HTB2::mDHFR F[3]- HygB/*	This Study
YSB458	*Matα/can1Δ::ste2pr-Sp_HIS5/lyp1Δ/his3Δ/leu2Δ/LYS1/ura3Δ/met15Δ/ NHP6A::mDHFR F[1,2]-natMX / HTB2::mDHFR F[3]- HygB*	This Study
YSB608	*Matα/ CIA2-myc::KanMX/ MMS19-GFP::HIS3/ Gal1p-CIA1*	This Study
YSB82	*Matα/ CIA2-HA::KanMX/ MMS19-TAP::HIS3*	This Study
YSB611	*Matα/***a** *Homozygote to: MMS19::mDHFR F[3]- HygB/ CIA2::mDHFR F[1,2]-natMX*	This Study
YSB61	*Matα/***a***/MMS19::mDHFR F[3]- HygB/ MMS19/ CIA2::mDHFR F[1,2]-natMX /CIA2*	[1]
YSB59	*Matα/***a***/CLN3::mDHFR F[3]- HygB/ CDC19::mDHFR F[1,2]-natMX*	[1]
YSB778	*Matα/ cia1(TS)::URA3-FLAG::natMX*	This Study
YSB778	*Mat**a** / Gal1p-CIA1::HIS3-FLAG::natMX*	This Study
YSB417	*Mat**a** cth1 Δ::URA3/ cth2 Δ:: natMX/ CIA2-HA::KanMX/ MMS19-TAP::HIS3*	This Study
YSB780	*Mat**a** ISA1-FLAG:: natMX / cth1 Δ::URA3/ cth2 Δ:: natMX/ CIA2-HA::KanMX/ MMS19-TAP::HIS3*	This Study

1. Tarassov K, Messier V, Landry CR, Radinovic S, Serna Molina MM, et al. (2008) An in vivo map of the yeast protein interactome. Science 320: 1465-1470.
2. Giaever G, Chu AM, Ni L, Connelly C, Riles L, et al. (2002) Functional profiling of the Saccharomyces cerevisiae genome. Nature 418: 387-391.
